# “Ingroup love” and “outgroup hate” in intergroup conflict between natural groups

**DOI:** 10.1016/j.jesp.2015.04.008

**Published:** 2015-09

**Authors:** Ori Weisel, Robert Böhm

**Affiliations:** a University of Nottingham, Nottingham, United Kingdom; b RWTH Aachen University, Aachen, Germany

**Keywords:** Intergroup conflict, Intragroup conflict, Ingroup love, Outgroup hate, Team games

## Abstract

We report on two studies investigating the motivations (“ingroup love” and “outgroup hate”) underlying individual participation in intergroup conflict between natural groups (fans of football clubs, supporters of political parties), by employing the *Intergroup Prisoner's Dilemma Maximizing-Difference* (IPD-MD) game. In this game group members can contribute to the ingroup (at a personal cost) and benefit ingroup members with or without harming members of an outgroup. Additionally, we devised a novel version of the IPD-MD in which the choice is between benefiting ingroup members with or without *helping* members of the outgroup. Our results show an overall reluctance to display outgroup hate by actively harming outgroup members, except when the outgroup was morality-based. More enmity between groups induced more outgroup hate only when it was operationalized as refraining from help.

## Highlights

• We experimentally examine intergroup conflict between natural groups.
• We compare behavior of groups with no/weak/strong enmity.
• Outgroup hate could be expressed by harm or by help avoidance.
• Enmity matters only for help avoidance, except when conflict is morality-based.
• The stronger the enmity, the more people chose to avoid helping the outgroup.

“*As far as I'm concerned they're bad people, just bad people.*”^1^

Schalke FC fan about Dortmund FC fans

There is a joke about an old man who was a lifelong fan of the local football club. When his doctor told him he was about to die he canceled his season ticket, and became a fan of another club, his old club's worst and most hated rival. When asked “why change clubs just before you die?” he replied “better one of them dying than one of us”.

Our society is marked by many forms of intergroup conflict. Wars between countries, clashes between ethnic and religious groups, long-lasting guerilla campaigns, and rivalries between street gangs or groups of sport fans have severe negative consequences to members of the participating groups, and sometimes also to un-involved bystanders (e.g., Cohen & Insko, 2008; Levitt & Venkatesh, 2000; McDonald, Navarrete, & Van Vugt, 2012). Despite being (by definition) an intergroup phenomenon, intergroup conflict is ultimately made possible by the actions of the individuals who take part in the conflict. These individuals often incur personal costs by participating in the conflict, but their precise motivation is ambiguous. The willingness to incur the cost of participation can be a result of a cooperative desire to help the ingroup (“ingroup love”), an aggressive/competitive motivation to hurt the outgroup or increase the gap between the groups (“outgroup hate”), or a combination of both (Allport, 1954; Brewer, 1999).^2^ Removing the ambiguity between ingroup love and outgroup hate as motivations for individual participation in intergroup conflict has been the focus of recent experimental work. The results suggest that ingroup love, rather than outgroup hate, is the main motivation at work (De Dreu, 2010; De Dreu et al., 2010; Halevy, Bornstein, & Sagiv, 2008; Halevy, Weisel, & Bornstein, 2012).

We extend this line of work in two ways. First, we examine behavior of members of natural groups, as opposed to the above-mentioned work which is based on laboratory games played between artificially created groups, members of which do not share any meaningful characteristics or history. In addition to increasing external validity, basing group membership on natural groups allows for the quasi-experimental manipulation of key characteristics of real intergroup conflicts, namely the degree of enmity between groups (Diekhof, Wittmer, & Reimers, 2014), and the extent to which the intergroup relations are based on similar or opposing moral convictions (Haidt, 2001; Parker & Janoff-Bulman, 2013).

Second, we allow participants to exhibit outgroup hate by not helping the opponent group, rather than only by directly harming it, a distinction that has been shown to make a behavioral difference (e.g., Greenwald & Pettigrew, 2014; Mummendey et al., 1992). Including the option to exhibit outgroup hate by help-avoidance allows us to test whether previous results, which stress the prevalence of ingroup love as the main motivation at play, are limited to cases where outgroup hate can be expressed by direct harm.

## Individual motivation in intergroup conflict: “ingroup love” and/or “outgroup hate”

Intergroup conflict is complex. Alongside the obvious conflict between groups, it often involves internal conflicts within each competing group. Individual group members face a tension between their self-interest and their group's welfare, and can also be motivated by concerns regarding the other group(s). The *Intergroup Prisoner's Dilemma* (IPD) game (Bornstein, 1992) is a model of intergroup conflict which simultaneously considers the intragroup and intergroup levels of conflict. Each group member decides whether (or how much) to contribute to the group's effort. Contribution is costly to the individual, beneficial to the ingroup, and harmful to the outgroup (see Table 1). The intragroup level of conflict is captured by the tension between individual and group interests: non-contribution maximizes the individual's payoff, but group payoffs are maximized when individuals contribute as much as possible. The intergroup level of conflict is captured by the tension between group and collective interests: while each group is best served by maximal contributions by its members, the most efficient outcome from the collective point of view, that of all individuals from both groups, is that no one contributes anything.

Despite the negative effect on collective welfare, there are substantial contributions to the group pool in the IPD. In some cases these contributions are considerably higher than those in a structurally equivalent single-group prisoner's dilemma game, in which contribution has no adverse effect on the outgroup (Bornstein & Ben-Yossef, 1994).^3^ Contribution in the IPD, as in the real world intergroup conflicts which it models, has two consequences: increasing the ingroup's welfare and decreasing the outgroup's welfare. Contribution is thus motivationally ambiguous and can be attributed either to ingroup love or to outgroup hate. The motivation for non-contribution is also not clear, but can be either plain selfishness or a reluctance to harm the outgroup.

In an attempt to behaviorally disentangle the motivational ambiguities in the IPD, the *Intergroup Prisoner's Dilemma-Maximizing Difference* (IPD-MD) game (Halevy et al., 2008) provides individuals with an additional option. They can choose between non-contribution, contribution to a between-group pool, which is costly to the individual, beneficial to the ingroup, and harmful to the outgroup (as in the IPD), or to a within-group pool which is also costly to the individual and beneficial to the ingroup, but does not affect the outgroup (see Table 1). Helping the ingroup in the IPD-MD is possible either with (between-group pool) or without (within-group pool) harming the outgroup. Harming the outgroup, however, is possible only by contributing to the between-group pool, so these contributions are rather clear indications of an aggressive and/or competitive motivation towards the outgroup (i.e., outgroup hate). Similarly, non-contribution is unambiguously selfish as it cannot be motivated by a reluctance to harm the outgroup or to increase collective welfare.

Halevy et al. (2008) compared behavior in the IPD and IPD-MD. Their results are clear-cut: while there were considerable contributions to the between-group pool in the IPD game (about 30%), there were hardly any in the IPD-MD game (about 6%). Additionally, overall contributions increased when players had the possibility to benefit the ingroup without necessarily harming the outgroup (i.e., in the IPD-MD). These results show an overwhelming preference for ingroup love over outgroup hate when players can behaviorally discriminate between the two motivations. Similar results have been obtained when comparing the IPD and the IPD-MD games in a repeated-game setting, even after an artificially created “history of conflict”, in which only the between-group pool was available for a certain number of repetitions before the within-group pool was introduced as a third option (Halevy, Weisel, & Bornstein, 2012).

### Minimal vs. natural groups and positive vs. negative externalities

The strong preference for ingroup love over outgroup hate in the IPD-MD is seemingly in contrast with at least one major theory of intergroup relations in social psychology — Social Identity Theory (SIT; Tajfel & Turner, 1979, 1986; see Hogg, Abrams, Otten, & Hinkle, 2004, for an overview). According to SIT, group members aim to maximize relative rather than absolute outcomes in comparison to outgroup members, in order to create positive distinctiveness. In the IPD-MD this implies using the between-group pool, which harms the outgroup while helping the ingroup, increasing the gap between the groups (in the ingroup's favor). The infrequent use of the between-group pool in previous laboratory experiments, however, shows that group members had little concern for their group's relative standing vis-à-vis the other group.

We propose that there are at least two reasons for this somewhat surprising result. First, previous research using the IPD-MD investigated very “minimal” groups. The artificial group categories had no meaning or prior history over and above the positive outcome interdependence of ingroup members in the game, such that participants' ties with their groups (“group commitment”; Ellemers, Spears, & Doosje, 2002) were rather weak. Furthermore, participants had no particular reason to evaluate the opposing group negatively, to feel threatened by it, or to view it as morally opposed. These are perhaps not the most favorable conditions for outgroup hate to emerge. In real world intergroup conflicts (e.g., between ethnic, religious, or political groups) group commitment is typically high, opposing groups are evaluated negatively, are often perceived as a severe threat for the ingroup's welfare, and conflict can have a strong moral dimension (Sherif & Sherif, 1969; see Riek, Mania, & Gaertner, 2006, for a meta-analysis). Therefore, the low levels of outgroup hate observed in previous studies may be the result of lack of group commitment or insufficient negative attitudes towards the opposing group (Kilduff, Elfenbein, & Staw, 2010). Brewer (1999) argued that ingroup love is a function of positive attitudes towards the ingroup, whereas outgroup hate is a function of negative attitudes towards the outgroup. In a similar vein, Cuddy, Fiske, & Glick (2007) claim that active harm towards another group is associated with low warmth stereotypes. Furthermore, Parker & Janoff-Bulman (2013) demonstrate that morality-based group identification is inductive to negative outgroup emotions.

Second, in the IPD-MD participants could display outgroup hate only by choosing the between-group pool, thereby actively imposing a negative externality on members of the outgroup. In many real world intergroup relations, however, outgroup hate may be shown rather indirectly, for instance by not hiring an outgroup applicant or avoiding to help an outgroup member in need. Here, outgroup hate is displayed passively by avoiding to impose a positive externality on outgroup members (see e.g., Cuddy et al., 2007, on the distinction between active and passive harm). Previous research suggests that the distinction between positive and negative outgroup externalities has behavioral implications. The positive–negative asymmetry in social discrimination (Buhl, 1999; Mummendey & Otten, 1998; Mummendey, Otten, Berger, & Kessler, 2000; Mummendey et al., 1992) postulates that intergroup discrimination emerges mostly in the allocation of positive, rather than negative, resources. Greenwald & Pettigrew (2014) (see also Banaji & Greenwald, 2013) review a host of laboratory and field experiments, and conclude that present-day discrimination in America is primarily a result of selectively helping members of advantaged groups, rather than harming members of disadvantaged groups. Given the central role that help-avoidance seems to play in intergroup discrimination, the low levels of outgroup hate in previous studies might be attributed to the focus on negative, rather than positive, externalities on the outgroup.

## The current research

To examine whether the infrequent use of outgroup hate in previous work is a result of groups' artificiality and/or the focus on negative externalities, we studied the behavior of members of natural groups—fans of football clubs (Study 1) and supporters of political parties (Study 2)—in settings where displays of outgroup hate were possible by either imposing a negative externality, or refraining from imposing a positive externality, on the outgroup.

### Degree of enmity and moral convictions among natural groups

The degree of enmity between natural groups is often relational (i.e., particular to specific pairs of groups). In sports, some teams merely compete in the same league, while others have long historic rivalries fueled by regular direct competition for titles or differences in fan bases' political preferences or religious identification.^4^ In politics, ideologically similar parties can engage in relatively “friendly” competition over voters, but ultimately cooperate and ally to form a coalition, while ideologically polarized parties often have fundamentally hostile and competitive relationships. Group members' psychological involvement and perceived stakes of competition with another group are determined by specific features that characterize the competition, and can be subjective, i.e., independent of the objective threat imposed by the other group or the extent to which its goals are incongruent with those of the own group (Kilduff et al., 2010).

Carefully selecting the specific natural groups in the studies allowed us to quasi-experimentally manipulate the degree of enmity between groups. The football clubs (Study 1) and political parties (Study 2) that define group membership were chosen such that cross-matching them (within each study) yields both weak- and strong-enmity pairs. Matching a group of fans (supporters) of a certain club (party) with another group of fans (supporters) of the same club (party) yields a no-enmity pair. In this case intergroup conflict stems only from the structure of the strategic interaction, as in previous research with artificial laboratory groups. Stronger degrees of enmity can lead to an increased motivation to participate in intergroup conflict, and are associated with negative attitudes towards the outgroup (Jackson, 2002; Riek et al., 2006; Sherif et al., 1961), which in turn lead to outgroup hate (Brewer, 1999). We therefore expect that more enmity will be related to an increased willingness to harm, or to avoid helping, the outgroup.

Another characteristic of natural groups, which is absent from artificial groups, is the degree to which they are morality-based. Members of morality-based groups share convictions about standards and principles on the distinction between right and wrong, as opposed to attitudes on matters of taste and preference (good or bad). Importantly, the degree to which shared moral convictions form the basis of a morality-based group depends on the existence of a salient outgroup holding opposing moral convictions (Haidt, 2001). For example, attitudes regarding the legality of abortion in the United States define distinct morality-based groups (pro-choice vs. pro-life) on the national level, where the respective outgroups are highly salient, but not in the context of, e.g., post graduate democrats, where pro-choice is nearly consensual.^5^

At the interpersonal level, disagreements on matters of morality are accompanied by strong emotions, intolerance, a desire for social/physical distance, lack of goodwill, and little regard for procedural safeguards (Parker & Janoff-Bulman, 2013). Relying on these results, Parker and Janoff-Bulman conjecture that this pattern should be relevant to intergroup relations as well, such that outgroup negativity will play a central role when groups are morality-based. They show that members of morality-based groups display more negative emotions towards (morally distinct) outgroups than members of non-morality-based groups. We incorporate morality-based groups into our investigation via the choice of parties in Study 2.

### A positive variant of the IPD-MD

To allow for displays of outgroup hate by help-avoidance, rather than by active harm, we introduce a positive variant of the IPD-MD. Similarly to the original IPD-MD, players can choose between non-contribution, an individually costly contribution to a within-group pool which benefits the ingroup and does not affect the outgroup, or an individually costly contribution to a between-group pool which affects both the ingroup and the outgroup. The difference is that the externality that contribution to the between-group pool imposes on the outgroup is negative in the original IPD-MD, but positive in the positive variant of the IPD-MD (see Table 1).^6^

The motivations associated with contribution to the within- and between-group pools in the positive variant are, to a large degree, opposite those in the previously explored IPD-MD. Collective welfare is maximized by contribution to the within-group pool in the original IPD-MD, but by contribution to the between-group pool in the positive variant. In both games contribution to either pool benefits the ingroup to the same degree, but while this can be done either with (between-group pool) or without (within-group pool) harming the outgroup in the original IPD-MD, in the positive variant the choice is between helping the outgroup (between-group pool) or avoiding to do so (within-group pool).

The positive variant is included in our study not only as a comparison to the original IPD-MD, but also as a realistic model of intergroup relations in itself. In some cases, group members face a choice between providing an exclusively internal public good (club good or local good; Cornes & Sandler, 1996), or one that can be enjoyed by the outgroup as well, as modeled by the positive variant.^7^

### Hypotheses

Hypotheses 1, 2, and 3 relate to both studies. Hypothesis 4 relates only to Study 2.

*Hypothesis 1* More overall contributions (regardless of pool) in the original IPD-MD and the positive variant of the IPD-MD than in the IPD. Previous research on intergroup conflict between artificial laboratory groups (mainly Halevy et al., 2008; Halevy, Weisel, & Bornstein, 2012) found more overall contributions in the original IPD-MD than the IPD. We expect this result to hold for the natural groups we examine as well, in both versions of the IPD-MD.

*Hypothesis 2* More outgroup hate when enmity is stronger. In both the original IPD-MD and the positive variant we expect that group members will be more prone to display outgroup hate the stronger the enmity between their club/party and that of the club/party supported by members of the other group. This hypothesis follows from the relation between degree of enmity and negative attitudes (Jackson, 2002; Riek et al., 2006; Sherif et al., 1961), and that of negative attitudes with outgroup hate (Brewer, 1999). A particular case of this hypothesis is that when there is no enmity at all between the groups, i.e., conflict stems only from the structure of the game, as in previous research with artificial laboratory groups, there will be less outgroup hate as compared to conflict between groups with at last some enmity.

*Hypothesis 3* More outgroup hate in the positive variant than in the original IPD-MD. Following the positive–negative asymmetry in social discrimination (e.g., Mummendey et al., 2000), we expect to find more outgroup hate in the positive variant, where it can be expressed by help-avoidance, than in the original IPD-MD, where it can be expressed by active harm.

*Hypothesis 4* More outgroup hate in interactions with a morality-based outgroup. In both the original IPD-MD and the positive variant we expect group members to be particularly prone to display outgroup hate when interacting with morality-based outgroups. Previous work found a similar pattern with respect to negative emotions (Parker & Janoff-Bulman, 2013).

## Study 1 — football fans

The groups we investigate in Study 1 are fans of four first-league (Bundesliga) German football clubs — Borussia Dortmund (BD), FC Schalke 04 (FCS), 1. FC Köln (FCK), and Bayer 04 Leverkusen (BL). Using fans of football clubs, and these teams in particular, has a few advantages. First, football is the most popular sport in Germany (arguably in the world). Many people identify as fans and are highly committed to their fan group (77% of Germans are interested in the German Bundesliga; Schnabel, 2009). The high level of interest is important because domain interest is a predictor of intergroup schadenfreude (pleasure at another's misfortune; Leach, Spears, Branscombe, & Doosje, 2003, demonstrated this phenomena among Dutch football fans). Second, cultural and socio-economic differences between the groups are largely avoided in comparison to other group categories, e.g., nationality, religion, or political affiliation.

**Fig. 1:**
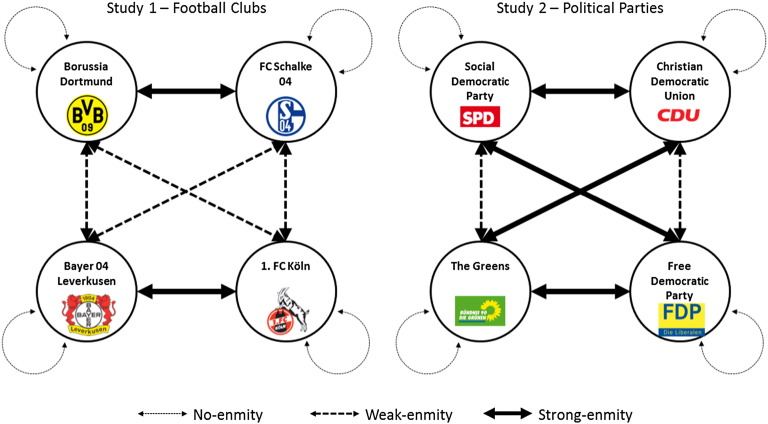
In study 1, the degree of enmity between opposing groups was manipulated by matching a group of fans of a given club with another group of fans of the same club (e.g., BD vs. BD; no-enmity); with a group of fans of a club with no special rivalry (e.g., BD vs. FCK; weak-enmity); or with a group of fans of a club with a strong historic rivalry (e.g., BD vs. FCS; strong-enmity). In study 2 the same was done for supporters of political parties.

Finally, by matching groups of fans according to the degree of enmity between their supported clubs, we can quasi-experimentally manipulate the degree of enmity with the opponent group. Considering the four clubs we investigate, BD and FCS have perhaps the strongest historic rivalry^8^ in the German Bundesliga (dubbed the “Ruhr Derby”, as both clubs are located in the Ruhr region in the German state of North Rhine-Westphalia), and BL and FCK have an established historic rivalry as well (the “Middle Rhine Derby”). Apart from these two pairs there is no special conflict between the teams, aside from that which arises from regular competition in the same league (e.g., there is no particular conflict between BD and BL; see Fig. 1 (left) and the Method section below for more details on matching and on the degree of enmity manipulation).

### Method

#### Participants and design

Three hundred ninety-five football fans completed an online study using EFS survey by Questback. A link to the study was posted on various web pages related to the four clubs (e.g., fan bulletin boards) and emailed to fan clubs. The completion rate was 64% (614 persons began the study). The study included an intergroup conflict game followed by a questionnaire. Participants' mean age was 35 years (*SD* = 12.46; completed by 275 participants) and 14% were female (completed by 274 participants). 31% of the participants had a university degree, 32% a high school degree, and 37% a lower, or no, academic qualification. Overall, participants were quite active fans: 34% attended all home games, 29% attended most games, and 30% attended some games in the current season.

We used a 3 (degree of enmity with the opponent group: none vs. weak vs. strong) × 3 (game: IPD vs. original IPD-MD vs. positive variant of IPD-MD) between-subjects design. Each participant was assigned with probability 1/9 to one of the conditions.^9^

#### Procedure

At the beginning of the online study participants were informed that upon its completion six of them will be chosen at random, and that only these six will be paid according to their decisions (which was indeed the case). Each participant was then informed that he/she is a member of a three-person group, together with two other participants who are fans of the same club; that their group will be matched with another three-person group, whose members are also fans of one particular club; and which club that is. The degree of enmity (none, weak, or strong) between the two groups was manipulated via the identities of the clubs supported by members of each group (see Fig. 1, left).

Participants were then told that they have three actions to choose from (two in the IPD); that their decision has financial consequences for themselves, their two group members, and the three members of the opposing group; and that the other participants have exactly the same options, all affecting each other's outcomes.

They could selfishly keep an endowment worth €40; contribute it to a between-group pool, which, in the IPD and original IPD-MD helps the ingroup and harms the outgroup, and in the positive variant of the IPD-MD helps the ingroup and the outgroup; or, in the IPD-MD games, contribute to a within-group pool, which helps the ingroup without affecting the outgroup.

The available actions and their monetary consequences are displayed in Table 1.^10^ The actions were not labeled or named (see Fig. S1 in the Supplementary Online Materials for a decision screen). After seeing a detailed example of possible choices and their consequences, participants chose the option that best suits their preferences. Finally, they completed a short questionnaire assessing their attitude towards fans of each of the four clubs and basic demographics (age, gender, education level). The study took about 10 min to complete.

*Dependent variables Decision in the game*. The main dependent variable was the choice between non-contribution or contribution to the between-group pool in the IPD, or between non-contribution, contribution to the between-group pool, or contribution to the within-group pool in the IPD-MD games. In the original IPD-MD contribution to the within-group pool indicates ingroup love and contribution to the between-group pool indicates outgroup hate. In the positive variant contribution to the within-group pool indicates outgroup hate and contribution to the between-group pool indicates ingroup love.

*Attitude towards fan groups.* The attitude towards each of the four fan groups was assessed by the following four items: “What is your general attitude regarding the fans of BD/FCS/FCK/BL?” (1 = very negative to 7 = very positive). The responses were used for a degree enmity manipulation check.^11^

**Fig. 2:**
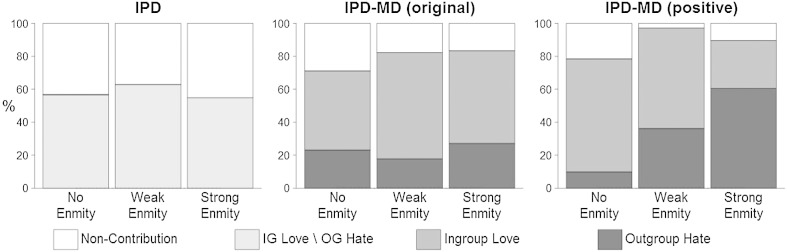
Study 1: Proportion of participants who chose non-contribution (to keep their endowment), ingroup love, or outgroup hate in each game (IPD, original IPD-MD and positive variant of IPD-MD) and for each degree of enmity with the opposing group (none, weak-enmity, strong-enmity). Ingroup love stands for the within-group pool in the original IPD-MD and for the between-group pool in the positive variant; outgroup hate stands for the between-group pool in the original IPD-MD, and for the within-group pool in the positive variant of the IPD-MD.

*Results*A manipulation check confirmed that the three degree of enmity conditions coincide with participants' attitude ratings towards the different fan groups (see Supplementary Online Materials). Fig. 2 shows the proportion of non-contribution and of contribution to each pool for each degree of enmity with the opponent group, separately for each game. We tested the effects of the game and the degree of enmity on participants' decisions with a generalized linear mixed effect model (with a logit link function), using the *lme4* package (Bates, Maechler, & Bolker, 2012) in the R environment (R Core Team, 2012). The specific fan group of the decision maker and that of the opposing group were modeled as random effects to control for their interrelated error terms (Pinheiro & Bates, 2000).

*Non-contribution vs. contribution*. We first analyzed the effect of the game (IPD vs. both versions of the IPD-MD) and of the degree of enmity with the opponent group on overall contributions (non-contribution vs. contribution to either pool).^12^ Regression coefficients, standard errors, 95% confidence intervals, and *p*-values are presented in Table 2 (top).^13^ As predicted, overall contributions in the IPD-MD games (83%) were significantly higher than in the IPD (58%; indicated by the IPD-MD predictor). Adding the interaction term to the model revealed that this was the case irrespective of the degree of enmity with the opponent group. This result supports Hypothesis 1, as well as previous research (Halevy et al., 2008; Halevy, Weisel, & Bornstein, 2012), by showing that contributions increase when they are not necessarily tied with harming the opponent group. These contribution rates are noticeably higher than those commonly observed in the literature, for example by Halevy et al. (2008) and Halevy, Weisel, & Bornstein (2012) who observe around 30% in the IPD and 35%–55% in the (original) IPD-MD.

*Ingroup love vs. outgroup hate*. Next, we turned our attention to the IPD-MD games. While the ingroup love pools (the within-group pool in the original IPD-MD, the between-group pool in the positive variant) attracted the majority of participants (56% and 53%, respectively), there were also substantial contributions to the outgroup hate pools (the between-group pool in the original IPD-MD, the within-group pool in the positive variant): 23% in the IPD-MD, and 35% in the positive variant (the difference is marginally significant, *p* = .077; Table 2, bottom). These figures are much higher than the 4–6% obtained by Halevy et al. (2008) and Halevy, Weisel, & Bornstein (2012).

A more interesting difference between the two versions of the IPD-MD is the effect of the degree of enmity with the opponent group. There was significantly more outgroup hate when facing a strong-enmity opponent, relative to facing a no-enmity opponent (strong predictor in bottom part of Table 2). However, adding the interaction terms to the regression equation shows that this effect is solely due to the positive variant (where even facing a weak-enmity opponent increased outgroup hate), and not at all to the original IPD-MD game, where the degree of enmity did not matter. Related Hypothesis 2 is supported only for the positive variant, and not for the original IPD-MD.

Two points are noteworthy. The first is that the relation between degree of enmity and outgroup hate in the positive variant is not accompanied by a similar relation between degree of enmity and overall contributions, suggesting a two-stage process. Group members first choose—independent of degree of enmity—whether to contribute or not; and only then (if applicable) they choose between ingroup love and outgroup hate. The latter choice is related to degree of enmity only in the positive variant. The second point is that in the positive variant the majority (60%) of participants facing a strong-enmity opponent group chose to display outgroup hate. In other words, outgroup hate was the predominant behavioral motivation of more than half of our participants when it involved withholding resources from a strong-enmity opponent.

## Study 2 — supporters of political parties

Study 2 was conducted with two main objectives in mind. It could be that the results in Study 1 are due to unique features of the groups we examined (fans of German football clubs). Therefore, the first objective was to extend, or possibly qualify, our results by examining another set of naturally occurring rivalrous groups. The second objective was to include morality-based (out)groups in our examination.

The groups we examine in Study 2 are based on political affiliation. Socio-demographic differences, which naturally exist between supporters of different parties, were mitigated by including only students in our sample.^14^ The degree of enmity was manipulated as in Study 1, by having each group composed of supporters of the same party. The core of the design, aimed at replicating and extending Study 1, is provided by supporters of the following four German mainstream parties: The Christian Democratic Union (CDU), The Social Democratic Party (SPD), The Free Democratic Party (FDP), and The Greens (GREENS). Two additional parties from the political extreme—The Left (LEFT) and the National Democratic Party (NPD)—were included to provide morality-based outgroups. The Left was later dropped from the analysis because supporters of the mainstream parties did not perceive it to be as morality-distinct as the NPD (see Supplementary Online Materials for more details).

Recent coalitions in Germany were formed, in various constellations, by the four mainstream parties in our study. The CDU and the SPD are the two largest parties, and the only ones with the pretense of forming and leading the coalition. The FDP is the “natural” coalition partner of the CDU (government partners in the German Bundestag in 1961–1966, 1982–1998, and 2009–2013), and GREENS is the “natural” coalition partner of the SPD (1998–2005). Given this structure we expected that the degree of enmity would be strong between parties from opposing sides of the German political divide (i.e., GREENS and SPD on one side, CDU and FDP on the other), and weak between natural coalition partners. The result is a very similar design to Study 1, with the exception that in Study 1 each fan group had one strong- and two weak-enmity outgroups, while in Study 2 each supporter group has one weak- and two strong-enmity outgroups (see Fig. 1, right).

While political preferences among mainstream parties in Germany surely involve moral aspects, the discourse is mild in comparison with, e.g., the United States. The mainstream parties are not as polarized, the relations among them are not as conflictual, and many consider them to have more in common than in separation. To include morality-based groups, we added two extreme parties, one from the far-right (the National Democratic Party of Germany, NPD) and the other from the far-left (the Left Party, LEFT) of the political spectrum.^15^ Among these two parties the NPD is clearly more controversial and more likely to be perceived as morally distinct by mainstream voters. Indeed, a manipulation check showed that the NPD is perceived as a morality-based outgroup by supporters of all four mainstream parties, whereas the LEFT is not. Accordingly, we omitted the LEFT from the analysis, and treated the NPD as the sole morality-based outgroup in our sample.^16^

*Method* The method of Study 2 was nearly identical to that of Study 1. Differences are highlighted below.

*Participants and design* Two thousand three hundred seventy-five students from three German universities (University of Erfurt, University of Jena, and RWTH Aachen University) responded to an email inviting them to take part in an online questionnaire with the possibility to earn money. One thousand five hundred and fifty were eventually included in our sample (*n_Erfurt_* = 279, *n_Jena_* = 832, *n_Aachen_* = 438, 1 missing value). The remaining 825 were excluded for the following reasons: 161 did not support any of the six parties they could choose from; 14 supported the NPD; 326 supported the LEFT; and 324 people were matched with supporters of the LEFT. Participants' mean age was 25 years (*SD* = 5.34) and 45% were female.

We used a 4 (degree of enmity with the opponent group: none vs. weak vs. strong vs. morality-based) × 3 (game: IPD vs. original IPD-MD vs. positive variant of IPD-MD) between-subjects design. Participants were assigned randomly to one of the three games, and there was an equal probability of being matched with supporters of each of the six parties in the initial design (including the LEFT). After removing the LEFT, the probability of being matched with one of the two strong-enmity outgroups was therefore double (2/5) that of being matched with the ingroup, a weak-enmity outgroup, or a morality-based outgroup (1/5 each).

*Procedure* At the beginning of the study participants were asked which party they supported, from a menu including CDU, SPD, FDP, GREENS, NPD, and the LEFT. They could also indicate that they do not support any of these parties, in which case they were thanked and did not proceed. Each participant was then informed that he/she belongs to a three-person group, with two other supporters of the same party; that their group will be matched with another three-person group, whose members support one particular party; and which party that is. The strategic situation was presented exactly as in Study 1. The questionnaire at the end assessed attitudes towards supporters of each of the six parties (and basic demographics).

**Fig. 3:**
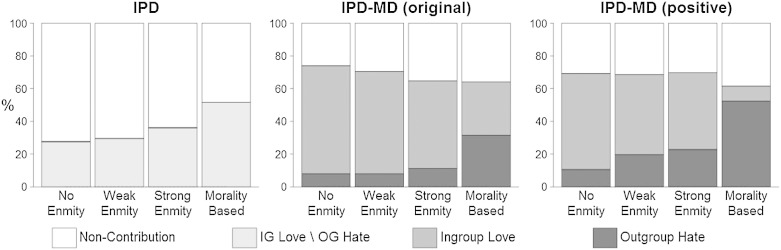
Study 2: Proportion of participants who chose non-contribution (to keep their endowment), ingroup love, or outgroup hate in each game (IPD, original IPD-MD and positive variant of IPD-MD) and for each degree of enmity with the opposing group (none, weak-enmity, strong-enmity, morality-based). Ingroup love stands for the within-group pool in the original IPD-MD and for the between-group pool in the positive variant; outgroup hate stands for the between-group pool in the original IPD-MD, and for the within-group pool in the positive variant of the IPD-MD.

*Results* A manipulation check confirmed that the three degree of enmity conditions coincide with participants' attitude ratings towards the different parties (see Supplementary Online Materials). Fig. 3 shows the proportion of non-contribution and of contribution to each pool for each degree of enmity with the opponent group, separately for each game. For the statistical analysis, we applied the same generalized linear mixed effect model as in Study 1 (Bates et al., 2012; Pinheiro & Bates, 2000; R Core Team, 2012).

*Non-contribution vs. contribution*. We first analyzed the effect of the degree of enmity with the opponent group and of the game (IPD vs. both versions of the IPD-MD) on overall contributions. Regression coefficients, standard errors, 95% confidence intervals, and significance levels are presented in Table 3 (top). Overall contributions in the IPD-MD games (68%) were significantly higher than in the IPD (36%), providing further support for Hypothesis 1. The interaction terms show that this effect is strongest for no-enmity, and is significantly weaker for strong-enmity and morality-based opponents (note the negative interaction coefficients for strong-enmity and morality-based opponents). The effect is also significantly weaker for morality-based than for strong-enmity opponents (*p* < 0.01; obtained by re-running the regression with strong-enmity as the reference level of the degree of enmity dummy variable).

The above pattern suggests that the difference in contributions between the IPD and IPD-MD games diminishes as conflict grows more severe. Still, contributions in the IPD-MD games are significantly higher than in the IPD for weak- (*p* < .001) and strong-enmity opponents (*p* < .001), and the difference is marginally significant for morality-based opponents (*p* = .070; *p*-values obtained by re-running the regression with different orders of dummy variables). These results can be interpreted as strengthening Study 1 in supporting Halevy et al. (2008) and Halevy, Weisel, & Bornstein (2012) by showing that for all degrees of enmity, and even for morality-based outgroups, contributions in the IPD are lower than in the IPD-MD games. At the same time, they add to Study 1 by showing that the magnitude of the tendency to increase overall contributions in the IPD-MD relative to IPD is not independent of the degree of enmity between the groups; most notably, it is reduced in interactions between groups with a morality-based outgroup.

*Ingroup love vs. outgroup hate*. Focusing on the IPD-MD games reveals that there was more outgroup hate in the positive variant than in the original IPD-MD (Table 3, bottom), confirming Hypothesis 3. There was more outgroup hate in strong-enmity than no-enmity, and even more with morality-based opponents. Adding the interaction terms reveals that the difference in outgroup hate between the original IPD-MD and the positive variant was largest for morality-based opponents, and not significant for no-enmity opponents. The model with interactions also shows that the difference between no- and strong-enmity was not significant in the original IPD-MD (Table 3), but was in the positive variant (*p* < 0.01, obtained by re-running the regression with the positive variant as the reference level), replicating the result from Study 1. As long as conflict does not have a strong moral aspect, the degree of enmity matters only in the positive variant, and not in the original IPD-MD. Thus, in both studies, Hypothesis 2 is supported only for the positive variant of the IPD-MD.

Morality-based groups showed the highest levels of outgroup hate in both the original IPD-MD and the positive variant, confirming Hypothesis 4 (Table 3, bottom). However, in the original IPD-MD, outgroup hate is not the prevalent choice even in conflict with a morality-based outgroup. Instead, there are practically identical levels of ingroup love and outgroup hate; about half of those choosing to contribute restrict the effect of their contribution to the ingroup and avoid harming outgroup members. This result is a strong demonstration of the reluctance many people have towards actively harming others, even if they are members of a negatively viewed and morally opposed outgroup.

**Discussion** We contribute to the social psychological literature on intergroup conflicts and its motivational underpinnings (e.g., Allport, 1954; De Dreu, 2010; Halevy et al., 2008) by examining behavior of members of natural groups—football fans and supporters of political parties—with varying degrees of enmity in three intergroup conflict games. Along with two previously established intergroup conflict games—the *Intergroup Prisoner's Dilemma* (IPD) game and the *Intergroup Prisoner's Dilemma-Maximizing Difference* (IPD-MD) game—we introduced a novel positive variant of the latter, which allows for expressions of outgroup hate by help-avoidance.

There are two main messages from our studies. The first is that the main result of Halevy et al. (2008), and Halevy, Weisel, & Bornstein (2012)—that much of the competitive behavior observed in the IPD is eliminated when an option to maximize absolute, rather than relative, ingroup gains is introduced—holds even for members of rivalrous groups. This was the case for both football fans and supporters of political parties. From a conflict resolution perspective this is a rather optimistic message, strengthening the assertion that “intergroup conflicts can be resolved by channeling group member's altruism towards internal group causes” (Halevy et al., 2008, p. 410). Even when conflict had a strong moral flavor, outgroup hate was reduced in the IPD-MD relative to the IPD, with only about half of the contributors opting for outgroup hate over ingroup love.^17^

The second message is not so optimistic. When given the chance to benefit a strong-enmity outgroup, and even more so a morality-based outgroup, many group members decline to do so. In these settings the desire to increase relative gains, or to simply harm the outgroup, plays an important role. This is unfortunate because of the loss of potential social gains, which is not different, in principle, from the losses associated with actively harming the outgroup. This result is particularly striking for morality-based groups, where 85% of those who chose to contribute in the positive variant of the IPD-MD did so without (costlessly) helping outgroup members.

The difference we observed between negative and positive displays of outgroup hate is in agreement with previous work on minimal groups, which has found more intergroup discrimination in the allocation of positive resources than negative resources (e.g., Buhl, 1999; Mummendey & Otten, 1998; Mummendey et al., 1992, 2000). Several explanations may account for this result. One possibility is a general tendency to evaluate negative events more negatively when they are due to commission rather than to omission (e.g., Spranca, Minsk, & Baron, 1991). In social interaction, discrimination may be perceived as less appropriate when it involves active harm of the outgroup than passive help-avoidance. Another option is that western societies, where most studies showing the positive–negative asymmetry were conducted (including the present study), are characterized by a promotion focus (concern for approaching positive events, rather than avoiding negative ones), which has been shown to crucially moderate the prevalence of discrimination in helping behavior (Sassenberg, Kessler, & Mummendey, 2003). Yet another possible explanation for the reluctance to help outgroup members has to do with status conferral. While harming outgroups decreases prestige but increases dominance, helping outgroups decreases both prestige and dominance, and is thus less attractive (Halevy, Chou, Cohen, & Livingston, 2012). Our results suggest that this status conferral pattern is relevant mainly for strong-enmity or morality-based opponent groups.

The degree of enmity between groups—the focus of the current investigation—is not the only characteristic of intergroup relations that might be relevant for outgroup hate. Another important characteristic is the relative deprivation/gratification of one group versus the other. Relative deprivation is particularly relevant; in minimal group settings it induces outgroup derogation (Moscatelli, Albarello, Prati, & Rubini, 2014) and outgroup hate in the IPD-MD game (Halevy, Chou, Cohen, & Bornstein, 2010). In the present context, however, relative deprivation/gratification is not trivial to assess. Straight forward measures like league standings or poll predictions can be misleading, as they fail to take into account differences in expectations (based on, e.g., previous league standings or current parliament seats) and resources (e.g., player salary, campaign budget). We tried to reduce the saliency of relative deprivation/gratification in Study 1 by timing data collection to be removed from direct matches between the clubs. In Study 2 this was obviously not possible. Given its relevance in minimal group settings, it is of interest to address the role of relative deprivation/gratification in natural group settings as well.

Our results on morality-based groups reflect positively on the conjecture that outgroup hate is particularly likely in morality-based conflict (Parker & Janoff-Bulman, 2013). We found this to be the case when outgroup hate could be displayed by directly harming the outgroup, and by refraining from help as well. These findings extend previous results, which rely on self-reported outgroup emotions, to actual behavior.

Moral-conflict aside, there was less outgroup hate in Study 2 (political parties) than in Study 1 (football). This might be due to the relatively mild political discourse in Germany, especially among the mainstreams parties, even when they are on different sides of the liberal-conservative political divide. Another reason might be that football fans in Study 1 were approached via club-specific web pages (e.g., fan bulletin boards), while participants in Study 2 were approached via general student subject pools and indicated their party affiliation after starting the survey. The result might be that our Study 1 sample is composed of fans with a relatively salient group affiliation. Importantly, the overall results (more outgroup hate in the positive variant than in the original IPD-MD, effect of degree of enmity on outgroup hate only in the positive variant), even without considering morality-based conflicts, are similar between Study 1 and Study 2.

An interesting route for further studies is to manipulate the way the intergroup interaction is perceived by framing the situation in different ways, for example by introducing different initial (default) allocations to the different pools. If manifestations of outgroup hate, or discrimination, can indeed be reduced merely by framing an interaction as involving active harm rather than passive help-avoidance, there are possible policy implications. For example, stressing the similar ultimate consequences of harm and help-avoidance via education or soft nudges (Thaler & Sunstein, 2008) can help reduce the negative effects associated with discrimination.

The newly developed positive variant of the IPD-MD has theoretical value beyond the scope of the current work. For example, according to the BIAS map framework (Cuddy et al., 2007) very specific stereotypes and emotions may give rise to either active or passive forms of discrimination. More generally, Greenwald and Pettigrew (2014) discuss at length the methods used in previous discrimination studies, and conclude that “…researchers need to add methods … that (a) distinguish ingroup-favorable from outgroup-hostile subject behavior” (p. 678). The combination of the original IPD-MD and the positive variant provides a method that nicely meets this requirement, and may provide a fresh approach to the study of intergroup prejudice and discrimination.

A better understanding of intergroup conflict is inevitably linked to a better understanding of individuals' motivation for participation in the conflict. Previous research suggested that ingroup love is the main motivation at play. We demonstrate that outgroup hate can also play an important role, depending on the degree of enmity between the groups, whether it involves imposing negative externalities or denying positive externalities, and whether conflict is morality-based.

## Figures and Tables

**Table 1:** Games, actions, and payoffs (Studies 1 and 2).

		Effect on					
		Ingroup member			Outgroup member		
Game	Action	1 (self)	2	3	1	2	3
IPD	Keep	+€40	0	0	0	0	0
	Between group pool	+€20	+€20	+€20	−€20	−€20	−€20

IPD-MD (original)	Keep	+€40	0	0	0	0	0
	Between group pool	+€20	+€20	+€20	−€20	−€20	−€20
	Within group pool	+€20	+€20	+€20	0	0	0

IPD-MD (positive)	Keep	+€40	0	0	0	0	0
	Between group pool	+€20	+€20	+€20	+€20	+€20	+€20
	Within group pool	+€20	+€20	+€20	0	0	0

Note: The table illustrates the effect an individual's action has on her own payoff, the payoff of each of two other ingroup members, and that of the three outgroup members. Each player's final payoff is determined by the combined effect of the six (three ingroup + three outgroup) players' actions.

**Table 2:** Generalized linear mixed effect models (Study 1 — football fans).

Effect of game and degree of enmity on overall contributions								
	Without interaction				With interaction			
Predictor	b	SE	95% CI		b	SE	95% CI	
Intercept	− 0.12	0.29	− 0.70	0.46	0.25	0.39	− 0.51	1.01
Game								
IPD (Ref)								
IPD-MD (both) (2)	1.39^⁎⁎⁎^	0.26	0.88	1.89	0.87^⁎^	0.43	0.02	1.72
Degree of enmity								
None (Ref)								
Weak (2)	0.71^⁎^	0.31	0.10	1.32	0.24	0.49	− 0.72	1.19
Strong (3)	0.45	0.29	− 0.12	1.02	− 0.09	0.48	− 1.04	0.86
Interaction								
Game (2) × Enmity (2)	–	–	–	–	0.74	0.64	− 0.53	2.00
Game (2) × Enmity (3)	–	–	–	–	0.83	0.61	− 0.36	2.03

Effect of game and degree of enmity on outgroup hate								

	Without interaction				With interaction			

Predictor	b	SE	95% CI		b	SE	95% CI	

Intercept	− 1.55^⁎⁎⁎^	0.33	− 2.18	− 0.91	− 0.73^⁎^	0.35	− 1.42	− 0.05
Game								
IPD-MD (Ref)								
IPD-MD positive (2)	0.51^†^	0.29	− 0.06	1.08	− 1.21^⁎^	0.59	− 2.37	− 0.05
Degree of enmity								
None (Ref)								
Weak (2)	0.40	0.38	− 0.35	1.14	− 0.55	0.53	− 1.60	0.49
Strong (3)	1.30^⁎⁎⁎^	0.35	0.61	2.00	0.00	0.49	− 0.95	0.96
Interaction								
Game (2) × Enmity (2)	–	–	–	–	1.97^⁎^	0.80	0.41	3.53
Game (2) × Enmity (3)	–	–	–	–	2.67^⁎⁎⁎^	0.76	1.19	4.15

b = regression coefficients; SE = standard errors; Ref = reference group; 95% CI = 95% confidence intervals (based on the estimated local curvature of the likelihood surface). En-dashes indicate that the variable was not included in the model. Note: All models considered the specific fan group (e.g., BD, FCS) of the decision maker and that of the opposing group as random effects. ^†^*p* < 0.1, ⁎ *p* < 0.05, ⁎⁎ *p* < 0.01, ⁎⁎⁎ *p* < 0.001

**Table 3:** Generalized linear mixed effect models (Study 2 — supporters of political parties).

Effect of game and degree of enmity on overall contributions								
	Without interaction				With interaction			
Predictor	b	SE	95% CI		b	SE	95% CI	
Intercept	− 0.69^⁎⁎⁎^	0.19	− 1.06	− 0.33	− 1.06^⁎⁎⁎^	0.24	− 1.53	− 0.58
Game								
IPD (Ref)								
IPD-MD (both) (2)	1.36^⁎⁎⁎^	0.11	1.13	1.58	1.95^⁎⁎⁎^	0.25	1.45	2.45
Degree of enmity								
None (Ref)								
Weak (2)	0.02	0.17	− 0.32	0.35	0.13	0.30	− 0.46	0.72
Strong (3)	0.04	0.14	− 0.24	0.32	0.43^†^	0.25	− 0.05	0.92
Morality-based (4)	0.12	0.17	− 0.21	0.46	1.08^⁎⁎⁎^	0.29	0.50	1.65
Interaction								
Game (2) × Enmity (2)	–	–	–	–	− 0.23	0.38	− 0.97	0.50
Game (2) × Enmity (3)	–	–	–	–	− 0.64^⁎^	0.31	− 1.25	− 0.03
Game (2) × Enmity (4)	–	–	–	–	− 1.50^⁎⁎⁎^	0.36	− 2.20	− 0.79

Effect of game and degree of enmity on outgroup hate								

	Without interaction				With interaction			

Predictor	b	SE	95% CI		b	SE	95% CI	

Intercept	− 2.51^⁎⁎⁎^	0.28	− 3.06	− 1.95	− 2.11^⁎⁎⁎^	0.37	− 2.84	− 1.38
Game								
IPD-MD (Ref)								
IPD-MD positive (2)	1.04^⁎⁎⁎^	0.19	0.65	1.42	0.40	0.50	− 0.58	1.37
Degree of enmity								
None (Ref)								
Weak (2)	0.52	0.33	− 0.13	1.17	0.05	0.55	− 1.03	1.12
Strong (3)	0.82^⁎⁎^	0.29	0.26	1.38	0.54	0.43	− 0.31	1.39
Morality-based (4)	2.78^⁎⁎⁎^	0.32	2.15	3.41	2.08^⁎⁎⁎^	0.46	1.18	2.97
Interaction								
Game (2) × Enmity (2)	–	–	–	–	0.75	0.69	− 0.61	2.10
Game (2) × Enmity (3)	–	–	–	–	0.45	0.57	− 0.67	1.57
Game (2) × Enmity (4)	–	–	–	–	1.38^⁎^	0.66	0.09	2.67

b = regression coefficients; SE = standard errors; Ref = reference group; 95% CI = 95% confidence intervals (based on the estimated local curvature of the likelihood surface). En-dashes indicate that the variable was not included in the model. Note: All models considered the specific party (e.g., CDU, SPD) of the decision maker and that of the opposing group as random effects. ^†^*p* < 0.1, ⁎ *p* < 0.05, ⁎⁎ *p* < 0.01, ⁎⁎⁎ *p* < 0.001

## Footnotes

1 The Story of the Revierderby (YouTube video), 2007.

2 Following, e.g., Brewer (1999), De Dreu (2010), De Dreu et al. (2010), Halevy et al. (2008), and Halevy et al. (2012), we use “ingroup love” and “outgroup hate” to describe specific behavioral motivations in the context of intergroup conflict. The terms do not necessarily carry the emotional connotation often associated with “love” and “hate”. Other authors use different terms, e.g., “ingroup favoritism” and “outgroup hostility” (Greenwald & Pettigrew, 2014), to convey similar concepts.

3 The higher contribution rate in the IPD as compared to an equivalent single group prisoner's dilemma game is in line with the intergroup conflict — intragroup cooperation/cohesion hypothesis (Benard & Doan, 2011; Bornstein, 2003), which claims that intragroup cooperation increases if the intragroup conflict is embedded in an intergroup conflict.

4 Some examples are the rivalry between the Boston Celtics and the Los Angeles Lakers, which is mainly a result of meeting in twelve NBA finals between 1959 and 2010; the game between the left-wing oriented FC St. Pauli and the right-wing oriented FC Hansa Rostock, dubbed the “political derby” (sometimes also the “fear derby”) in German football, and often a catalyst for violent clashes between fan groups; and the Glasgow Derby between Celtic F.C. and Rangers F.C. in Scottish Football which lies at the heart of Scottish “Sectarianism”, dividing fans along religious (catholic vs. protestant) and political (conservatism vs. socialism) lines.

5 85% of post graduate democrats define themselves as “pro-choice” (Ertelt, 2011).

6 The positive variant of the IPD-MD is similar to a *Nested Social Dilemma* (NSD; Wit & Kerr, 2002) in that it distinguishes between two (personally costly) pro-social actions, one benefiting only the ingroup and the other benefiting the larger collective (irrespective of group membership). In a NSD, however, the collectively optimal action is costly from the ingroup's point of view, whereas it is costless in the positive variant of the IPD-MD.

7 For example, a local lake-cleaning project has no effect on neighboring districts/countries, but a river-cleaning project also helps downstream communities.

8 Note that ‘rivalry’ is not identical to ‘enmity’. While ‘rivalry’ refers to a state of competition between individuals or groups, ‘enmity’ is a feeling of active hostility that often accompanies rivalry.

9 There was a probability of 1/3 of being assigned to each of the three “degree of enmity” conditions. Since each club had two “weak enmity” clubs associated with it, the probability of being matched with each of them was 1/6.

10 To avoid negative outcomes, €50 were added to participants' payoff in the IPD and original IPD-MD. For similarity, €10 were added to participants' payoff in the positive variant of the IPD-MD, although negative outcomes were not possible in this game.

11 We used these items as a proxy for the degree of enmity, rather than a more direct measure, because they are applicable to one's own group as well as to other groups (which is necessary to have a comparable measure for the no-enmity condition).

12 The result of this analysis does not change qualitatively when separately comparing the IPD to the original IPD-MD or to the positive variant.

13 *p*-Values in the table refer to two-tailed tests; unless stated otherwise, we refer to *p*-values below 0.05 as indicating a significant difference.

14 A possible concern is that students might not have well-formed political opinions and preferences. However, we conducted the study during the nine days leading to the 2013 general national elections, when involvement in politics is maximal. When asked to indicate which party they support (by choosing a party from a menu), 91.1% of those who began the online study were able to do so.

15 The NPD is considered neo-Nazi and has borderline legitimacy in German politics. There is a current attempt to ban it from German politics, after a previous attempt failed in 2003 (Hamann, 2013). The LEFT is an indirect successor of the Socialist Unity Party of Germany that ruled the German Democratic Republic (East Germany) until 1989. Although the fourth largest party in the Bundestag, it has never been part of the government in unified Germany, and for many Germans it serves as a direct reminder of what they consider an unfortunate part of German history.

16 See Supplementary Online Materials for details on the manipulation check and for an analysis that includes the LEFT.

17 As intergroup relations are affected by social norms that vary across cultural settings and over time, these findings may be subject to historical and cultural changes (Gergen, 1973). Outgroup hate may play a different role in settings other than modern-day Germany.
