# Supplementary material for: “Ingroup love” and “outgroup hate” in intergroup conflict between natural groups
Source: J Exp Soc Psychol. 2015 Sep;60:110–20. doi: 10.1016/j.jesp.2015.04.008 (PMC4518042; doi:10.1016/j.jesp.2015.04.008)
Supplement: Supplementary file 1 — Supplementary material. [file mmc1.doc]

Supplementary materials

Study 1 – degree 0f enmity manipulation check

Table S1 shows the average attitude scores of each fan group towards fans of each of the four clubs. As expected, in all four fan groups participants had the most positive attitude towards fans of their own club, and the most negative attitude towards fans of their strong-enmity club. We pooled the two weak-enmity clubs, and performed paired-samples *t*-tests to test whether the differences in attitudes are significant. The tests revealed that for each of the four fan groups, the average attitude towards fans of one’s own club (no-enmity) was significantly more positive than towards fans of weak-enmity clubs, which in turn was significantly more positive than towards fans of the strong-enmity club (*p* < .05 for all comparisons). Not surprisingly, these differences were also significant when aggregating the data of the four groups.

Study 2 – degree 0f enmity manipulation check

Table S2 shows the average attitude scores among supporters of the four mainstream parties, as well as attitudes towards the NPD. We expected that participants from each of the four supporter groups would have the most positive attitude towards supporters of their own party, that the attitude towards the weak-enmity party (i.e., the natural coalition partner) would be more positive than towards each of the strong-enmity parties (i.e., the coalition opponents), and that attitudes towards the morality-based enmity party (i.e., NPD) would be the most negative. This was indeed the case for all four parties. Pooling the two strong-enmity parties, paired-samples *t*-tests reveal that for each of the four supporter groups, the average attitude towards supporters of one’s own party was significantly more positive than towards supporters of the weak-enmity party, which was significantly more positive than towards supporters of the strong-enmity parties, which in turn was more positive than towards supporters of the morality-based enmity party (*p* < .001 for all comparisons). These differences were obviously also significant when aggregating the data of the four parties.

Study 2 - morality manipulation check

**Method.** We ran an online survey among a sample of 104 students from RWTH Aachen University, recruited from the same subject pool used for the main part of Study 2 (which included student samples from Jena and Erfurt in addition to Aachen; see *Method* section). Each participant indicated which party he/she supports from a menu of six parties which included the four mainstream parties, the LEFT, and the NPD. Participants were asked to which degree they felt that each of the other five parties builds on similar/opposing moral principles as the party supported by themselves, on a seven-point scale ranging from “Opposing moral principles” to “Identical moral principles”.[[1]](#footnote-2)

**Results.** Of the 104 respondents, 10 did not support any of the parties, 7 supported the LEFT, and no one supported the NPD. Since we were interested mainly in the perceptions of supporters of the four mainstream parties, we omitted the 7 LEFT supporters, as well as the 10 non-supporters, and ended up with 87 observations. For each person, we aggregated responses to the three mainstream parties other than the supported one. Paired-samples *t*-tests revealed that the NPD was perceived as morally distinct from the mainstream parties among supporters of all mainstream parties (CDU: *t*(29) = 10.26, *p* < .001; GREENS: *t*(18) = 9.06, *p* < .001; FDP: *t*(8) = 4.59, *p* = .002; SPD: *t*(28) = 13.10, *p* < .001. The LEFT, however, was perceived as morally distinct only by supporters of the CDU (*t*(29) = 9.48, *p* < .001) and the FDP (*t*(8) = 6.37, *p* < .001) but not by supporters of the GREENS (*t*(18) = 0.89, p = .386) and the SPD (*t*(28) = -1.07, *p* = .293). Another important difference between the NPD and the LEFT is that the large majority of respondents (71 out of 87; 82%) chose the extreme low response (“Opposing moral principles”) when asked about the NPD, as compared to only 16 out of 87 who chose this response when asked about the LEFT. These results led us to omit the LEFT from our analysis, and treat the NPD as the sole morality-based outgroup in our sample.

Study 2 - analysis including the LEFT

Figure S2 shows the proportion of non-contribution and of contribution to each pool for each of the enmity conditions, and for the LEFT and the NPD as opponent groups as well, separately for each game. The only differences between Figure S2 and Figure 4 in the main text is that the latter does not include data pertaining to the LEFT, which was excluded from the main analysis, and that the NPD column in Figure 5 is labeled “Morality Based” in Figure 4.

The Figure and accompanying analysis (see Table S3) suggest that the pattern of ingroup love and outgroup hate triggered by having the LEFT as an opponent is distinct from that of the morality-based NPD, and similar to the mainstream parties. The LEFT did not invoke more outgroup hate than the no-enmity baseline in the IPD-MD, but did in the positive variant, and it invoked less outgroup hate than the NPD in both games (see Table S3).

| **Table S1.** Attitudes towards different fan groups (Study 1) | | | | | |
| --- | --- | --- | --- | --- | --- |
|  |  |  | | | |
|  |  | Attitude towards fans of | | | |
| Reference club |  | Own  club | Weak-enmity  club | | Strong-enmity club |
|  |  | BD | BL | FCK | FCS |
| Borussia Dortmund (BD) |  | 6.52  (0.70) | 3.35  (1.02) | 4.19  (1.39) | 1.98  (1.15) |
|  |  | FCS | BL | FCK | BD |
| FC Schalke 04 (FCS) |  | 6.49  (0.72) | 3.84  (0.94) | 2.97  (1.47) | 2.19  (1.25) |
|  |  | FCK | BD | FCS | BL |
| 1. FC Köln  (FCK) |  | 5.71  (1.49) | 4.69  (1.42) | 3.53  (1.47) | 2.53  (1.27) |
|  |  | BL | BD | FCS | FCK |
| Bayer 04 Leverkusen (BL) |  | 6.70  (0.48) | 4.10  (1.29) | 4.50  (1.51) | 3.20  (2.10) |
| *Mean* |  | *6.31*  *(1.02)* | *3.77*  *(1.04)* | | *2.22*  *(1.28)* |
| Attitudes of “Reference club” fans towards fans of their own club, their weak-enmity clubs, and their strong-enmity club, on a 1 (very negative) to 7 (very positive) scale. Standard deviations are in parentheses. | | | | | |

| **Table S2.** Attitudes towards different supporter groups (Study 2) | | | | | |  |
| --- | --- | --- | --- | --- | --- | --- |
|  |  |  | | | |  |
|  |  | Attitude towards supporters of | | | | |
| Reference party |  | Own  party | Weak-enmity party | Strong-enmity  party | | Morality-based party |
|  |  | GRUENE | SPD | CDU | FDP | NPD |
| GRUENE |  | 5.90  (0.82) | 4.94  (1.05) | 3.51  (1.18) | 2.86  (1.11) | 1.22  (0.66) |
|  |  | CDU | FDP | GRUENE | SPD | NPD |
| CDU |  | 5.68  (0.89) | 4.20  (1.13) | 3.70  (1.32) | 4.13  (1.03) | 1.47  (0.98) |
|  |  | FDP | CDU | GRUENE | SPD | NPD |
| FDP |  | 5.46  (1.12) | 4.82  (1.25) | 3.57  (1.42) | 3.93  (1.08) | 1.43  (0.93) |
|  |  | SPD | GRUENE | CDU | FDP | NPD |
| SPD |  | 5.73  (0.86) | 4.79  (1.24) | 3.58  (1.19) | 2.96  (1.23) | 1.27  (0.75) |
| *Mean* |  | *5.74*  *(0.89)* | *4.65*  *(1.19)* | *3.49*  *(1.05)* | | *1.33*  *(0.83 )* |
| Attitudes of “Reference party” supporters towards supporters of their own party, their weak-enmity party, their strong-enmity parties, and the NPD, on a 1 (very negative) to 7 (very positive) scale. Standard deviations are in parentheses. | | | | | | |

| **Table S3.** Generalized linear mixed effect models (Study 2 – Supporters of political parties – including the LEFT) | | | | | | | | | | | | | | | | |
| --- | --- | --- | --- | --- | --- | --- | --- | --- | --- | --- | --- | --- | --- | --- | --- | --- |
|  | | | | | | | | | | | | | | | | |
| Effect of *Game* and *Degree of enmity* on *overall contributions* | | | | | | | | | | | | | | | | |
|  |  | Without interaction | | | | | | |  | With interaction | | | | | | |
| Predictor |  | b |  | SE |  | 95% CI | | |  | b |  | SE |  | 95% CI | | |
| Intercept |  | -0.72 | *** | 0.17 |  | -1.06 |  | -0.38 |  | -1.05 | *** | 0.23 |  | -1.50 |  | -0.59 |
| Game |  |  |  |  |  |  |  |  |  |  |  |  |  |  |  |  |
| IPD (Ref) |  |  |  |  |  |  |  |  |  |  |  |  |  |  |  |  |
| IPD-MD (both) (2) |  | 1.41 | *** | 0.11 |  | 1.21 |  | 1.62 |  | 1.93 | *** | 0.25 |  | 1.44 |  | 2.43 |
| Degree of enmity |  |  |  |  |  |  |  |  |  |  |  |  |  |  |  |  |
| None (Ref) |  |  |  |  |  |  |  |  |  |  |  |  |  |  |  |  |
| Weak (2) |  | 0.01 |  | 0.17 |  | -0.33 |  | 0.35 |  | 0.13 |  | 0.30 |  | -0.46 |  | 0.72 |
| Strong (3) |  | 0.03 |  | 0.14 |  | -0.25 |  | 0.32 |  | 0.42 | † | 0.25 |  | -0.06 |  | 0.91 |
| The LEFT |  | 0.66 | *** | 0.17 |  | 0.32 |  | 1.01 |  | 0.77 | ** | 0.29 |  | 0.20 |  | 1.33 |
| NPD |  | 0.12 |  | 0.17 |  | -0.22 |  | 0.45 |  | 1.07 | *** | 0.29 |  | 0.50 |  | 1.64 |
| Interaction |  |  |  |  |  |  |  |  |  |  |  |  |  |  |  |  |
| Game (2) × Enmity (2) |  | --- |  | --- |  | --- |  | --- |  | -0.22 |  | 0.37 |  | -0.95 |  | 0.51 |
| Game (2) × Enmity (3) |  | --- |  | --- |  | --- |  | --- |  | -0.63 | * | 0.31 |  | -1.23 |  | -0.02 |
| Game (2) × Enmity (4) |  | --- |  | --- |  | --- |  | --- |  | -0.15 |  | 0.37 |  | -0.88 |  | 0.57 |
| Game (2) × Enmity (5) |  | --- |  | --- |  | --- |  | --- |  | -1.48 | *** | 0.36 |  | -2.18 |  | -0.78 |
|  |  |  |  |  |  |  |  |  |  |  |  |  |  |  |  |  |
| Effect of *Game* and *Degree of enmity* on *outgroup hate* | | | | | | | | | | | | | | | | |
|  |  | Without interaction | | | | | | |  | With interaction | | | | | | |
| Predictor |  | b |  | SE |  | 95% CI | | |  | b |  | SE |  | 95% CI | | |
| Intercept |  | -2.61 | *** | 0.28 |  | -3.16 |  | -2.06 |  | -2.11 | *** | 0.37 |  | -2.84 |  | -1.38 |
| Game |  |  |  |  |  |  |  |  |  |  |  |  |  |  |  |  |
| IPD-MD (Ref) |  |  |  |  |  |  |  |  |  |  |  |  |  |  |  |  |
| IPD-MD positive (2) |  | 1.19 | *** | 0.18 |  | 0.84 |  | 1.53 |  | 0.40 |  | 0.50 |  | -0.58 |  | 1.37 |
| Degree of enmity |  |  |  |  |  |  |  |  |  |  |  |  |  |  |  |  |
| None (Ref) |  |  |  |  |  |  |  |  |  |  |  |  |  |  |  |  |
| Weak (2) |  | 0.52 |  | 0.33 |  | -0.13 |  | 1.18 |  | 0.05 |  | 0.55 |  | -1.03 |  | 1.12 |
| Strong (3) |  | 0.83 | ** | 0.29 |  | 0.27 |  | 1.39 |  | 0.54 |  | 0.43 |  | -0.31 |  | 1.39 |
| The LEFT (4) |  | 0.64 | * | 0.31 |  | 0.04 |  | 1.25 |  | -0.35 |  | 0.54 |  | -1.42 |  | 0.71 |
| NPD (5) |  | 2.82 | *** | 0.32 |  | 2.18 |  | 3.45 |  | 2.08 | *** | 0.46 |  | 1.18 |  | 2.97 |
| Interaction |  |  |  |  |  |  |  |  |  |  |  |  |  |  |  |  |
| Game (2) × Enmity (2) |  | --- |  | --- |  | --- |  | --- |  | 0.75 |  | 0.69 |  | -0.61 |  | 2.10 |
| Game (2) × Enmity (3) |  | --- |  | --- |  | --- |  | --- |  | 0.45 |  | 0.57 |  | -0.67 |  | 1.57 |
| Game (2) × Enmity (4) |  |  |  |  |  |  |  |  |  | 1.46 | * | 0.67 |  | 0.15 |  | 2.77 |
| Game (2) × Enmity (5) |  | --- |  | --- |  | --- |  | --- |  | 1.38 | * | 0.66 |  | 0.09 |  | 2.67 |
| b = regression coefficients; SE = standard errors; Ref = reference group; 95% CI = 95% confidence intervals (based on the estimated local curvature of the likelihood surface). Dashes indicate that the variable was not included in the model.  Note: All models considered the specific party (e.g., CDU, SPD) of the decision maker and that of the opposing group as random effects.  ^†^ *p* < 0.1, * *p* < 0.05, ** *p* < 0.01, *** *p* < 0.001 | | | | | | | | | | | | | | | | |

**Figure Captions**

**Figure S1.** The original IPD-MD game as seen on-screen by fans of BD matched with fans of FCS. The options for the positive variant of the IPD-MD game were the same except that the payoffs to the members of the other group in option 3 are +20 € instead of -20 €. The IPD game included only the left and right options (without the middle option). Group affiliations were made salient by the logos of the respective teams.

**Figure S2.** Study 2: Proportion of participants who chose non-contribution (to keep their endowment), ingroup love, or outgroup hate in each game (IPD, original IPD-MD and positive variant of IPD-MD), for each degree of enmity with the opposing group (none, weak-enmity, strong-enmity) and for the two non-mainstreatm partied (NPD and the LEFT) in the initial design. ingroup love stands for the within-group pool in the original IPD-MD and for the between-group pool in the positive variant; outgroup hate stands for the between-group pool in the original IPD-MD, and for the within-group pool in the positive variant of the IPD-MD.

**Figure S1.**


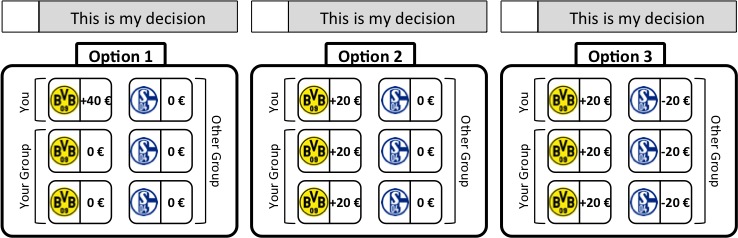


**Figure S2.**


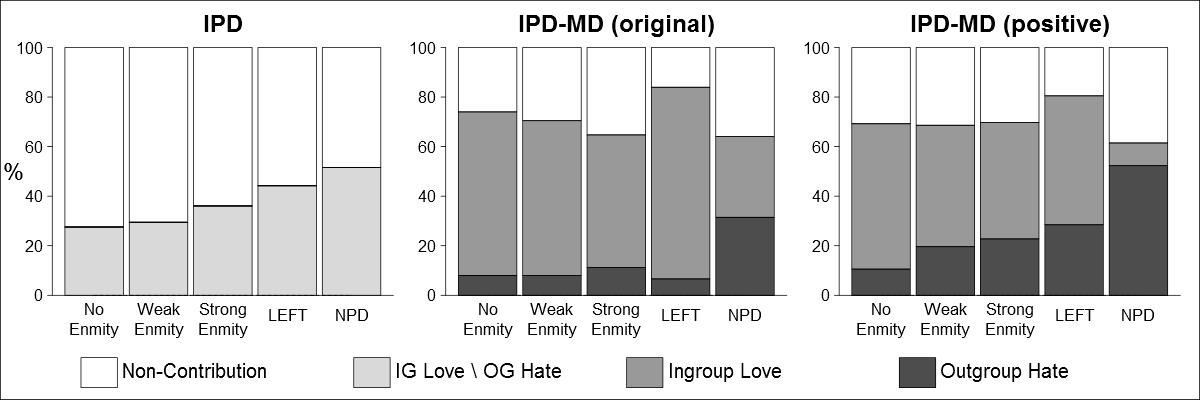


1. This straight forward item, high on face validity, was chosen because existing measures of moral identification (e.g., Aquino & Reed II, 2002; Iyer, Koleva, Graham, Ditto, & Haidt, 2012) are constructed to cluster people according to the moral foundations underlying their groups, but not necessarily to detect to which degree people *perceive* their own group as being similar/different from other groups. [↑](#footnote-ref-2)
